# Identification of a 3-Gene Prognostic Index for Papillary Thyroid Carcinoma

**DOI:** 10.3389/fmolb.2022.807931

**Published:** 2022-03-16

**Authors:** Lin-Kun Zhong, Xing-Yan Deng, Fei Shen, Wen-Song Cai, Jian-Hua Feng, Xiao-Xiong Gan, Shan Jiang, Chi-Zhuai Liu, Ming-Guang Zhang, Jiang-Wei Deng, Bing-Xing Zheng, Xiao-Zhang Xie, Li-Qing Ning, Hui Huang, Shan-Shan Chen, Jian-Hang Miao, Bo Xu

**Affiliations:** ^1^ Department of General Surgery, Zhongshan City People’s Hospital, Zhongshan, China; ^2^ Thyroid, Vascular Surgery Department, Maoming People’s Hospital, Maoming, China; ^3^ Department of Thyroid Surgery, Guangzhou First People’s Hospital, School of Medicine, South China University of Technology, Guangzhou, China; ^4^ Reproductive Medicine Center, Boai Hsopital of Zhongshan, Zhongshan, China; ^5^ Department of Intensive Care Medicine, Zhongshan City People’s Hospital, Zhongshan, China

**Keywords:** PTC, SVM diagnostic model, COX analysis, accurate diagnosis, prognostic evaluation

## Abstract

The accurate determination of the risk of cancer recurrence is a critical unmet need in managing thyroid cancer (TC). Although numerous studies have successfully demonstrated the use of high throughput molecular diagnostics in TC prediction, it has not been successfully applied in routine clinical use, particularly in Chinese patients. In our study, we objective to screen for characteristic genes specific to PTC and establish an accurate model for diagnosis and prognostic evaluation of PTC. We screen the differentially expressed genes by Python 3.6 in The Cancer Genome Atlas (TCGA) database. We discovered a three-gene signature Gap junction protein beta 4 (GJB4), Ripply transcriptional repressor 3 (RIPPLY3), and Adrenoceptor alpha 1B (ADRA1B) that had a statistically significant difference. Then we used Gene Expression Omnibus (GEO) database to establish a diagnostic and prognostic model to verify the three-gene signature. For experimental validation, immunohistochemistry in tissue microarrays showed that thyroid samples’ proteins expressed by this three-gene are differentially expressed. Our protocol discovered a robust three-gene signature that can distinguish prognosis, which will have daily clinical application.

## Background

Thyroid cancer (TC) is the most common malignant tumour in the endocrine system (The Lancet, 2017), whose most popular type is papillary thyroid carcinoma (PTC), accounting for 80–90% of all thyroid malignancies (Schneider and Chen, 2013; Kennedy and Robinson, 2016). If timely detection, diagnosis, and evaluation can be achieved during the early stages of PTC, coupled with the development of corresponding surgical methods, the patient’s follow-up treatment, disease surveillance, and prognosis will significantly improve. Therefore, it is of great importance to study the early screening, diagnosis, and prognosis of PTC.

Currently, the main clinical diagnostics for TC include high-resolution ultrasonography (US) and fine-needle aspiration (FNA), while FNA is the safest and most reliable test that can provide a definitive preoperative diagnosis of malignancy (Zheng et al., 2015; Ko et al., 2017). However, the sensitivity and specificity of FNA are reported to be 68–98% and 56–100%, respectively. This led to an increased rate of uncertain outcomes, underwent unnecessary diagnostic surgery, and received lifelong thyroid hormone replacement therapy with associated surgical complications. Preoperative molecular analysis using a panel of genetic alterations would overcome the limitation of FNA diagnosis (Muzza et al., 2020). Molecular markers have become a potential tool for TC management to distinguish benign from malignant lesions, predict aggressive biology, prognosis, recurrence, and identify novel therapeutic targets (Nylén et al., 2020).

Recently, with the development of genome sequencing technologies, more and more accumulating evidence has revealed that tumour biomarkers, including protein-coding genes, non-coding RNAs and immune genes, are informative for cancer detection and prognosis classification (Zhong et al., 2020; Gan et al., 2021). mRNAs have a great potential in physiological and pathological processes and predict the prognosis of various types of tumour patients (Feng et al., 2019; Tschirdewahn et al., 2019). Therefore, the dysregulated expression or mutation of RNA may be a promising predictor of poor prognosis in PTC. Thus, mRNAs’ dysregulated expression or mutation may be a promising predictor of poor prognosis in PTC.

The accurate determination of cancer diagnosis and treatment risk is a significant unmet need in PTC management. Patients and physicians must weigh the benefits of currently available therapies against the potential morbidity of these treatments. Herein we screen for characteristic genes specific to PTC and establish and validate an accurate model for PTC diagnosis and prognostic evaluation.

## Methods

### Patients and Tissue Samples

Tissue microarrays (TMA) of human TC (IWLT-N-58T53 TC-1503) involved in this experiment and research were purchased from Wuhan Aiwei Biological Technology Co. LTD., along with the detailed clinical information. It included 29 cases of PTC and 29 cases of para-cancer tissue. Of the 29 patients, 21 were female (aged 24–66 years), and eight were male (aged 27–60 years).

### Gene-Expression Data Sets

The gene expression and clinical data used for modelling were derived from TCGA (http://www.cbioportal.org/datasets), which contained gene expression data from 568 samples and clinical information from 516 samples. From The Cancer Genome Atlas (TCGA) database, clinical information was screened via the Cancer Type Detailed PTC parameter, of which a total of 399 samples were found. In these 399 PTC patients, 395 cases had RNA-seq data, of which 52 had para-cancer tissue data creating a total number of 447 RNA-seq data points. The gene expression data used to validate our model came from GSE27155 (Giordano et al., 2005; Giordano et al., 2006) of the GEO database (https://www.ncbi.nlm.nih.gov/geo/). The differentially expressed (DE) mRNAs between normal and PTC samples were assessed using the R Studio. software program (RStudio version 1.1.463; http://www.rproject.org), and the R package, Limma. log2FC (fold change) > 2 and *p*-value < 0.05 were considered for subsequent analyses (Gong et al., 2019). The project/collection had a total of 99 samples: four from normal patients, 10 cases of follicular adenomas, 13 cases of follicular thyroid carcinomas, 7 cases of eosinophilic thyroid adenomas, 8 cases of thyroid carcinomas, 51 cases of PTC, 4 cases of anaplastic thyroid carcinomas, and 2 cases of medullary thyroid carcinomas. In this study, we selected the 4 cases of normal patients and 51 cases of PTC to analyze.

### Feature Selection Methods

Python 3.6 was utilized to screen TCGA expression data for DE genes. The processing steps were as follows: Delete genes with an average expression value of less than 10 reads, which are considered to be genes of no research value in survival differences. Judge whether or not there is a significant difference at *p* < 0.05 between the two comparison groups using the SciPy package (https://www.scipy.org/) to perform *t*-test on the different study groups. Calculate the fold change value difference between groups by taking the mean value of different groupings.

**FIGURE 1:**
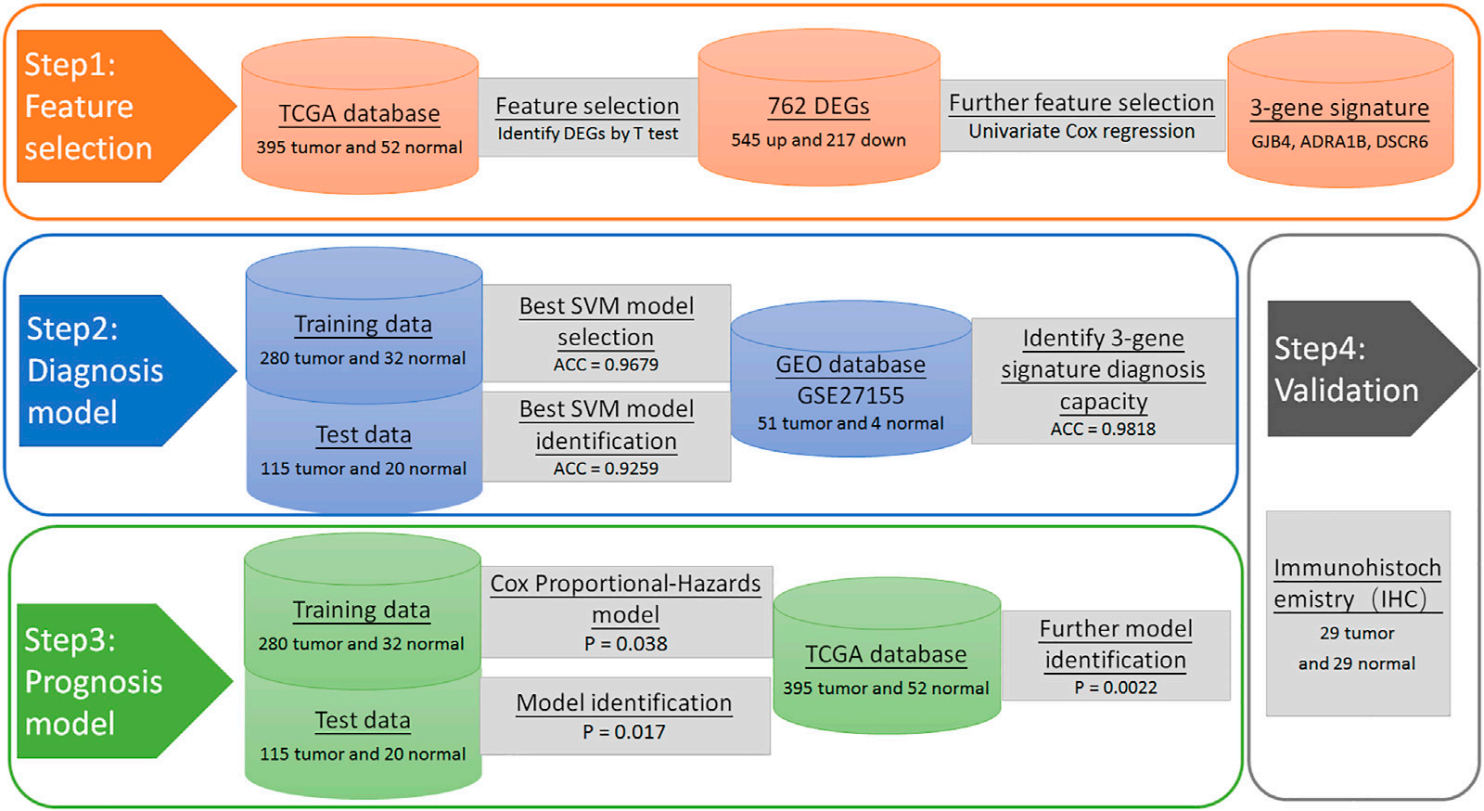
Flowchart of PTC prognostic signatures generation and validation procedures.

To find genes for use in modelling, we screened for characteristic genes that significantly affected PTC survival. The R 3.6 software was used to perform a univariate cox regression analysis between DE genes and clinical data (time, status) in 395 patients (Gill, 1992). Genes with a hazard ratio (HR) greater than 1, or less than 1, and a Wald test *p*-value of less than 0.05 were genes that significantly affected PTC survival. Therefore, selected these genes as characteristic genes for use in establishing a diagnostic model. We summarize the selection process in Figure 1.

### Establishing the Diagnosis and Prognosis Model

This study used the sklearn package (http://scikit-learn.org) provided in Python 3.6 to establish a Support vector machine (SVM) model to differentiate between cancer and non-cancer. Use the SVM classifier model to explore the optimal three-gene signature prognosis model. Based on the univariate Cox regression analysis of the selected characteristic genes, we established a prognostic model to calculate a patient’s prognosis by calculating their ‘RiskScore’ (Xiong et al., 2017). According to a set threshold (HR > 1 or HR < 1, *p* < 0.05), three-gene (Table 2) were found to be significantly associated with overall survival.

To test the diagnostic predictive power of the three-gene signature that we selected, we randomized TCGA PTC patients into a training set (312 samples, 70%) and a test set (135 samples, 30%). The training set was used for 10% cross-validation. The optimal parameters of the final model were (“C”: 1, “gamma”: 1,000, “kernel”: “rbf”), with the final average accuracy being 0.9263 (Standard Deviation: ± 0.0117). The average accuracy of our best model using the training set was 0.9679. To verify the effectiveness of this model, we used the best model predictions that gave an average accuracy of 0.9259. In addition, to verify the diagnostic predictive power of our three-gene signature, we also used the three-gene in GSE27155 and established an SVM model in the same way. Due to the small number of negative samples, we chose to use the 3-fold cross-validation and pre-determined optimal parameters of the model (“C”: 15, “gamma”: 1, “kernel”: “rbf”). This gave an accuracy of 0.9464 (SD: ± 0.0430).

### Microarray Preprocessing

Briefly, after deparaffinization in xylene and rehydration with graded concentrations of alcohol to distilled water, the TMA slides were washed in Tris-buffered saline with 0.1% Tween 20 (TBST), the slides were incubated with the primary antibody against GJB4 (1:10, Abcam, A9888), RIPPLY3 (1:75, Sigma-Aldrich, HPA055541), ADRA1B (1:50, Abcam, ab84405) at 4 °C overnight. After washing three times in TBST, the specifically bound secondary antibody was detected with the DAKO EnVision detection System (Dako Diagnostics, Switzerland). Immunostaining scores were independently performed by two experienced pathologists who did not know the patient’s clinical pathology data and the immediate clinical outcome. The staining intensity was scored as negative (1), weak (2), moderate (3) or strong (4). The staining extent was scored as 1 (≤10%), 2 (11–50%), 3 (51–75%) or 4 (>75%). A total expression score was calculated by multiplying the staining intensity score with that of the staining extent. ≤ 8 points were considered as low expression. Otherwise, it is considered as a high expression. Histological classification of the samples, stained with hematoxylin and eosin, was performed by two independent clinical pathologists.

### Functional Enrichment Gene Ontology Analysis

GO functional annotation pathway enrichments were performed in R using the “clusterProfiler” package, and *P* adjusted (FDR) < 0.05 was statistically significant.

### Construction of the Protein-Protein Interaction Network

The DE mRNA were imported into the STRING database (https://string-db.org/) (UniProt Consortium, 2010) to construct a PPI network. The network analysis plug-in in Cytoscape software was used to analyze network topological features to screen the hub nodes in the PPI network (Saito et al., 2012). Degree centrality denotes several direct connections of a node to all other nodes in the network.

### Data Analysis

Statistical analysis was performed using R 3.4.0 (https://www.r-project.org/), Python 3.6 (https://www.python.org/) and Graphpad Prism version 7.0 (GraphPad Software). A two-tailed Student’s t-test was used for comparisons between two independent groups. This study used the sklearn package (http://scikit-learn.org) provided in Python 3.6 to establish an SVM model to differentiate between cancer and non-cancer. All statistical analyses were two-sided. *p* < 0.05 was defined as indicating statistical significance (Ge et al., 2019).

## Results

### Bioinformatics Analysis Was Used to Screen Differentially Expressed Genes

**FIGURE 2:**
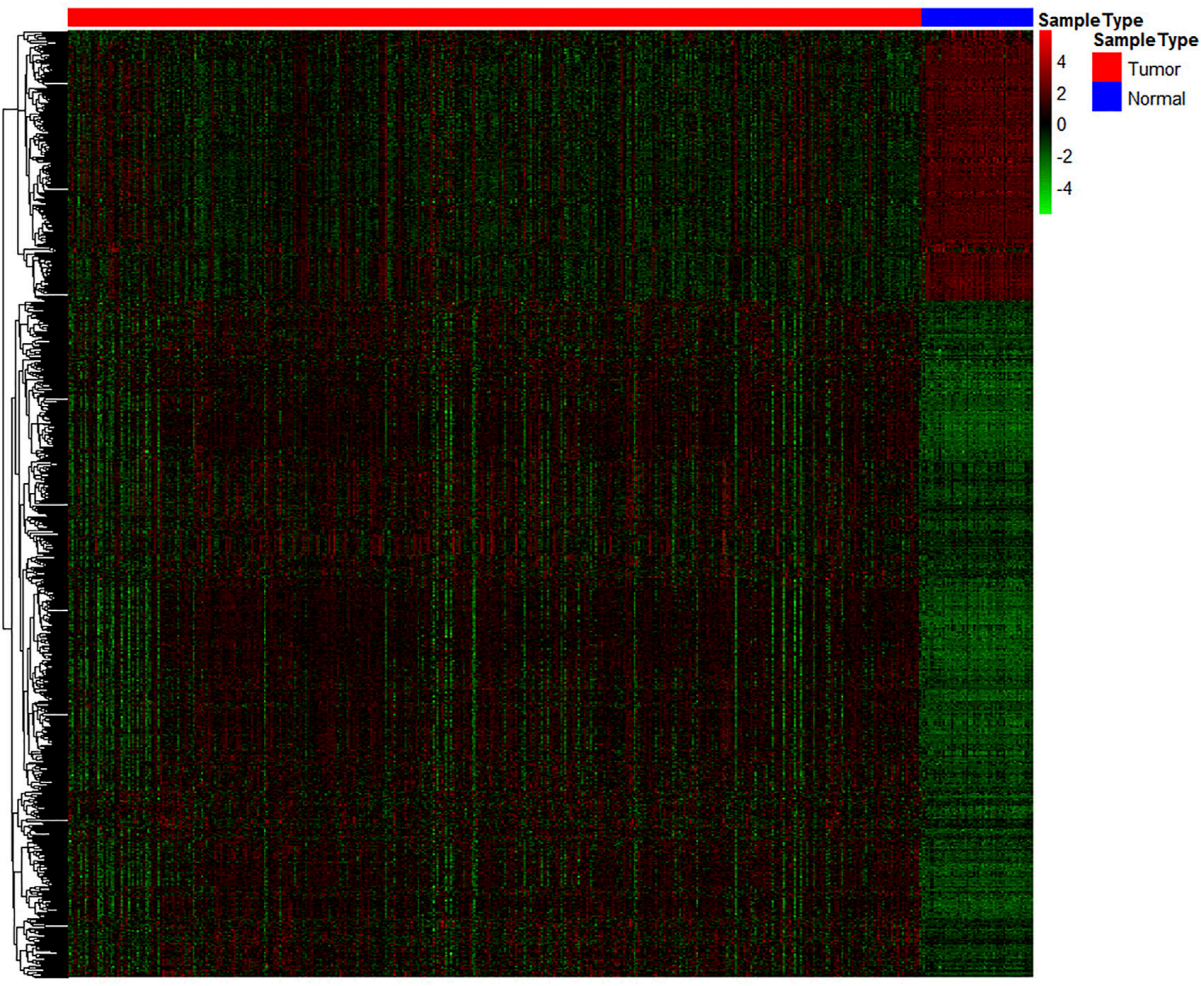
Heatmap of significantly differentially expressed genes. Each row represents a separate gene, each column represents a separate sample, a gradient from green to red indicates a low to high level of expression, and the samples are clustered from two types of tissue: normal tissue (green) and cancer tissue (red).

A total of 20,531 genes were screened from TCGA. After deleting genes with a mean expression of less than 10, 15,370 genes remained. The number of DE genes between tumour tissue and adjacent tissues was 762, of which 545 genes were upregulated, and 217 genes were down-regulated. The expression values of the DE genes were converted by log10 and displayed by heatmap. As seen from the heatmap (Figure 2), there is a significant difference between the tumour and the normal tissue, indicating that the results identified in this study are credible. The top 10 significantly upregulated and the top 10 significantly downregulated DE mRNA are displayed in Table 1.

**TABLE 1:** The top 10 upregulated and downregulated DE mRNA genes.

Type	Genes	LogFC	p value
Up-regulated	ARHGAP36	8.894666584	<0.001
	DMBX1	8.212911341	<0.001
	SLC18A3	8.071324334	<0.001
	TRY6	7.77283886	<0.001
	TMPRSS6	7.625107474	<0.001
	PRSS1	7.59111566	<0.001
	MMP13	7.567785968	<0.001
	KLK6	7.468232832	<0.001
	LOC400794	7.390954657	<0.001
	GABRB2	7.299817152	<0.001
Down-regulated	KCNA1	−4.139432844	<0.001
	TFF3	−3.811991634	<0.001
	LRP1B	−3.692660909	<0.001
	RELN	−3.629457676	<0.001
	IPCEF1	−3.521246594	<0.001
	ZNF804B	−3.519727733	<0.001
	CNTN5	−3.507769597	<0.001
	AGR3	−3.492012695	<0.001
	VIT	−3.43067668	<0.001
	FAM180B	−3.414101394	<0.001

DE,diferentially expressed;FC,fold change.

### Cox Regression Analysis Was Used to Screen Characteristic Genes

Through univariate Cox regression analysis, a hazard ratio was calculated for each gene according to the set threshold (HR > 1 or HR < 1, *p* < 0.05), To screen-specific biomarkers with accurate diagnostic ability. There were three genes found to be significantly related to overall survival (Table 2). These genes were GJB4, RIPPLY3, and ADRA1B. These three genes will be used as feature genes for subsequent modelling, and the specific information of genes is shown in Table 4.

**TABLE 2:** Univariate Cox regression analysis results.

Gene symbol	Beta	HR (95% CI)	*p*. value
GJB4	−0.057	0.94 (0.91–0.98)	0.0066
ADRA1B	−0.021	0.98 (0.96–0.99)	0.0067
RIPPLY3	−0.11	0.9 (0.81–0.99)	0.0360

### Establishing and Validate the Cox Prognostic Model

**FIGURE 3:**
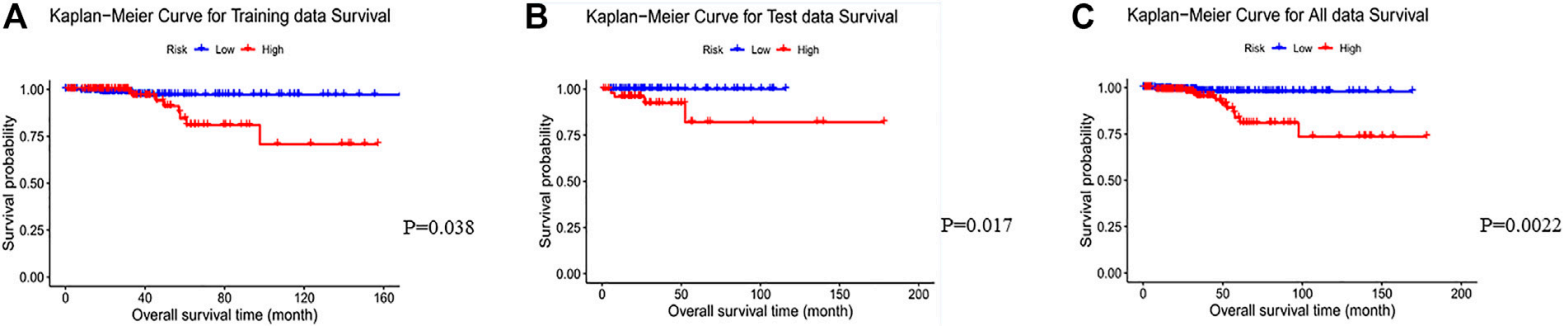
Kaplan-Meier curves for the low- and high-risk groups separated by the Risk-Score of the 3-gene signature in the TCGA PTC data. The blue line represents the patients with low risk and the others represent patients with high risk. Significant differences in overall survival between the two groups were analyzed by log-rank test. **(A**) Kaplan-Meier curves for training data survival; **(B)**Kaplan-Meier curves for test data; **(C)** Kaplan-Meier curves for all data.

**FIGURE 4:**
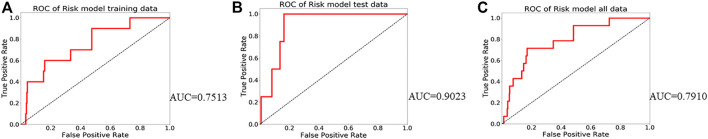
Receiver operating characteristic curves (ROC) for the prognosis models. **(A)** ROC fited based on training data; **(B)** ROC fited based on test data; **(C)** ROC fited based on all TCGA PTC data.

To test the prognostic prediction ability of the screened three-gene signature, we calculated the Risk-Score of each patient in the TCGA training set by using the established prognostic model. The risk assessment score formula was as follows: risk score= (-0.057×expression value of GJB4) + (−0.021×expression value of ADRA1B) + (-0.110×expression value of RIPPLY3). Table 3 Then the patients were ranked according to risk-score, and the risk-score median (−0.7766) was taken as the threshold. The 312 patients were divided into two groups, 156 patients with low risk and 156 patients with high risk. Of these 312 persons, 280 had survival information (OS), of which 156 were low risk, and 124 were high risk. Kaplan-Meier survival curves and ROC curves were used to examine the predictive power of three-gene biomarkers. Kaplan-Meier survival curves showed that the survival rate in the high-risk group was significantly lower than that in the low-risk group (log-rank *p* value = 0.038), Figure 3. The ROC curve was shown in Figure 4, and the AUC value was 0.7513, indicating that the three-gene signatures had a certain prognostic predictive ability. To further verify the prognostic capability of the three-gene signatures, we calculated the risk score of the patients in the test set and the PTC patient data set of the whole TCGA respectively and divided the patients into high-risk and low-risk patients’ groups according to the same threshold (−0.7766), Table 4. The Kaplan-Meier survival curves for both data sets showed significantly lower survival rates in the high-risk group than in the low-risk group (log-rank *p* value = 0.017 and 0.0022), with AUC values of 0.9023 and 0.7910, respectively. The training set and the results of two confirmations showed that the three-gene biomarkers screened had a strong prognostic capability.

**TABLE 3:** Differential expression information of characteristic genes.

Gene symbol	mRNA description	logFC	*P*	UP DOWN
GJB4	gap junction protein beta 4	4.0572	<0.001	UP
ADRA1B	adrenoceptor alpha 1B	2.4496	<0.001	UP
RIPPLY3	ripply transcriptional repressor 3	2.1379	<0.001	UP

FC,fold change.

**TABLE 4:** Survival analysis sample.

Data set	High risk	Low risk	High risk for OS	Low risk for OS	*p* value
Training set	156	156	156	124	0.038
Test set	72	63	52	63	0.017
All data	228	219	176	219	0.002

### Immunohistochemical Verification Results of Characteristic Gene Tissue Microarray

**FIGURE 5:**
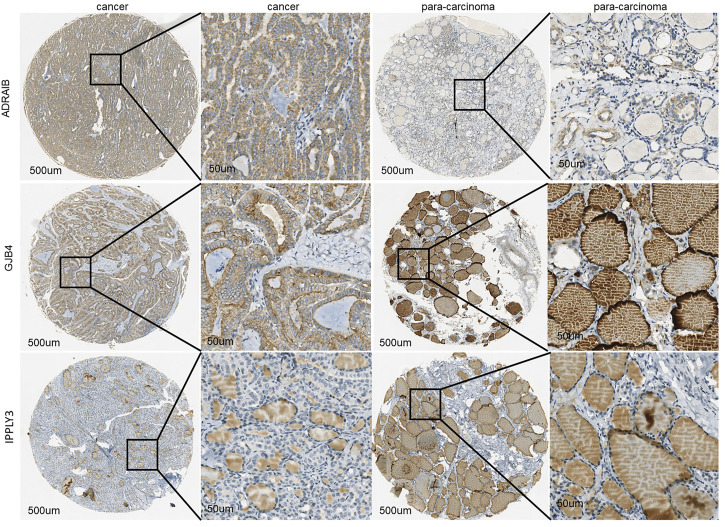
The immunohistochemical results of GJB4, RIPPLY3, and ADRA1B characteristic gene tissue chips indicated that the expression in tumor tissues was significantly higher than that in adjacent tissues.

To further verify the protein expression level of three-gene signatures in PTC tissue and analyze its relationship with clinicopathological features, immunohistochemistry was used to detect the protein expression level of tri-factor in 29 PTC tissue chips. The results of this study showed that the protein expression levels of GJB4 and ADRA1B in cancer cells were significantly higher than those in paired para-cancer cells (*p* < 0.05) (Figure 5), and the protein expression level of RIPPLY3 was not significant difference between the cancer cells and the para-cancer cells.

### GO Enrichment Analyses and Construction of the PPI Network

**FIGURE 6:**
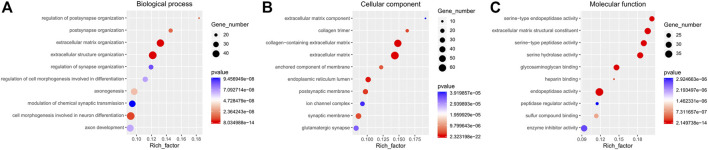
The GO enrichment analyses. **(A)**. Biological process; **(B)**. Cellular component; **(C)**. Molecular function.

**FIGURE 7:**
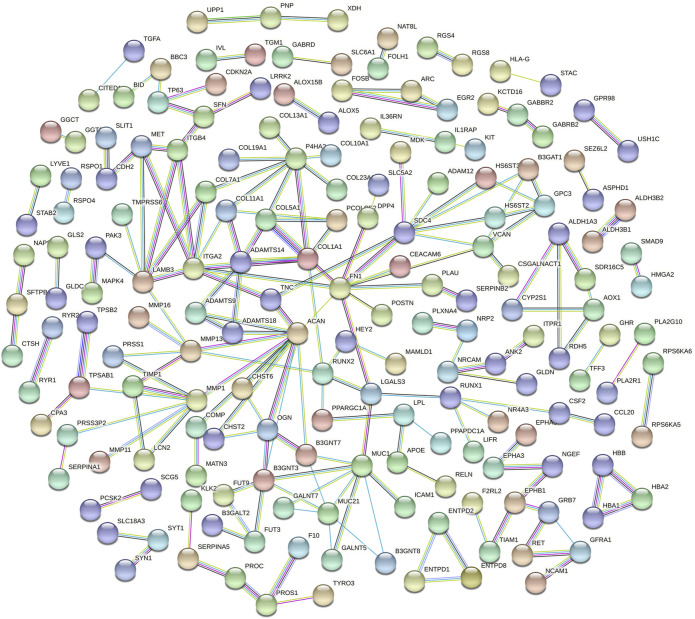
The PPI networks of DE mRNA.

Functional enrichment GO analyses were performed to investigate the underlying mechanisms of the DE mRNA genes’ prognostic effects. Our results demonstrate that the DE mRNA genes are linked with activating pathways, such as tumour development and progression regulation, based on GO analysis of three cohorts (Figure 6). PPI network reveal that the potential connection between key mRNA genes (Figure 7).

## Discussion

In our study, the results above clearly demonstrate that the three-gene signatures we screened for and selected have a strong ability to distinguish between cancerous and non-cancerous samples. The genes signature include GJB4, RIPPLY3, and ADRA1B. It was reported that GJB4 and ADRA1B genes play an essential role in developing many malignant tumours. It has been shown that GJB4 is involved in tumorigenesis and may act as a tumour promoter, Wang et al. (Wang et al., 2019) indicated that miR-492 promoted cancer progression by targeting GJB4 and was a novel biomarker for bladder cancer. Liu et al. (Liu et al., 2019) showed that GJB4 was highly expressed in gastric cancer tissues and cells, the high expression of GJB4 was significantly correlated with the overall survival of gastric cancer patients, and the cell proliferation and migration of gastric cancer cells were significantly inhibited by knockout GJB4. At the same time, targeting GJB4 may be exploited as a modality for improving lung cancer therapy had been proved (Lin et al., 2019). The ADRA1B gene is a member of the adrenergic receptor alpha 1 (ADRA1) subfamily, which also includes ADRA1A and ADRA1D, and has been shown to promote the development of cancer in the epinephrine cell pathway. Adrenergic receptor antagonists have also been shown to be useful in the treatment of various types of cancer, including prostate and breast cancer (Freudenberger et al., 2006; Harris et al., 2007). In present, no studies have been reported on the relationship between GJB4 and ADRA1B gene in TC. However, our study demonstrated that the GJB4 and ADRA1B genes may not be able to promote the thyroid cancer progression and development, and it may even be a protective gene.

Although no direct studies have proved that RIPPLY3 (also known as DSCR6) is closely related to PTC or other malignant tumours, current studies have found that the RIPPLY3 gene plays a role in developing the pharynx and its derivatives in vertebrates (Tsuchiya et al., 2018). Li et al. (Li et al., 2013) showed that RIPPLY3 is closely associated with Down syndrome (DS). Studies have found that people with DS have an increased risk of thyroid disease (mainly autoimmune), with a lifetime prevalence of between 13 and 63% (AlAaraj et al., 2019). These results suggest that RIPPLY3 may affect the development of the thyroid gland, and its abnormal expression may lead to the occurrence and development of PTC. The GO enrichment analysis revealed that the chief pathways regulated the cell-molecular function and the enzyme activity. Previous studies have demonstrated the gene effect on thyroid cell function and cell morphology (Yu et al., 2017; Rudzińska et al., 2019).

At present, the preoperative diagnosis of PTC is still mainly FNA. However, according to the Bethesda grading standard, the proportion of FNA diagnosis results is suspicious or uncertain is 3–18% (Misiakos et al., 2016). In the era of precision therapy, we need an accurate diagnosis, which requires an accurate prognosis. To find prognostic molecular markers of PTC, this study obtained the gene expression characteristic of tumor prognosis through TCGA to screen characteristic genes and carry out an effective risk assessment of tumour prognosis.

However, our study has some limitations, the most important one is the limited number of patients in our database group confined to the limitation to TCGA、GEO. There is still a lack of large sample data sets and clinical samples to verify the accuracy of the three-signature prognosis model. Also, we should further investigate the correlation between the three gene expression levels and the clinicopathological features. Fortunately, gene sequencing technology is gradually maturing and becoming faster and less expensive. We will continue to collect cases of TC tissue to verify our signature further. Furthermore, although we believe that the three-gene signature is promising in selecting patients who will benefit from the three-gene prognostic model, its significant value still needs to be verified in prospective studies.

## Conclusion

This study screened for DE genes (GJB4, RIPPLY3, ADRA1B) that were significantly related to the diagnosis and prognosis of PTC. The three-gene diagnostic model could accurately predict the occurrence of PTC and guide prognosis.

## Data Availability

The original contributions presented in the study are included in the article/supplementary materials, further inquiries can be directed to the corresponding authors.

## References

[B1] AlAaraj N. Soliman A. T. Itani M. Khalil A. De Sanctis V. (2019). Prevalence of Thyroid Dysfunctions in Infants and Children with Down Syndrome (DS) and the Effect of Thyroxine Treatment on Linear Growth and Weight Gain in Treated Subjects versus DS Subjects with normal Thyroid Function: a Controlled Study. Acta Biomed. 90 (8-S), 36–42. 10.23750/abm.v90i8-S.8503 PMC7233681 31544805

[B2] Feng G. Ma H.-M. Huang H.-B. Li Y.-W. Zhang P. Huang J.-J. (2019). Overexpression of COL5A1 Promotes Tumor Progression and Metastasis and Correlates with Poor Survival of Patients with clear Cell Renal Cell Carcinoma. Cmar Vol. 11, 1263–1274. 10.2147/cmar.s188216 PMC6369854 30799953

[B3] Freudenberger R. S. Kim J. Tawfik I. Sonnenberg F. A. (2006). Optimal Medical Therapy Is superior to Transplantation for the Treatment of Class I, II, and III Heart Failure: a Decision Analytic Approach. Circulation 114 (1 Suppl. l), I62–I66. 10.1161/CIRCULATIONAHA.105.001412 16820647

[B4] Gan X. Guo M. Chen Z. Li Y. Shen F. Feng J. (2021). Development and Validation of a Three-Immune-Related Gene Signature Prognostic Risk Model in Papillary Thyroid Carcinoma. J. Endocrinol. Invest. 44 (10), 2153–2163. 10.1007/s40618-021-01514-7 33620716

[B5] Ge W. Cai W. Bai R. Hu W. Wu D. Zheng S. (2019). A Novel 4-gene Prognostic Signature for Hypermutated Colorectal Cancer. Cmar Vol. 11, 1985–1996. 10.2147/cmar.s190963 PMC6407520 30881123

[B6] Gill R. D. (1992). Multistate Life‐tables and Regression Models. Math. Popul. Stud. 3 (4), 259–276. 10.1080/08898489209525345 12343718

[B7] Giordano T. J. Au A. Y. Kuick R. Thomas D. G. Rhodes D. R. Wilhelm K. G. Jr. (2006). Delineation, Functional Validation, and Bioinformatic Evaluation of Gene Expression in Thyroid Follicular Carcinomas with the PAX8-PPARG Translocation. Clin. Cancer Res. 12 (7 Pt 1), 1983–1993. 10.1158/1078-0432.CCR-05-2039 16609007

[B8] Giordano T. J. Kuick R. Thomas D. G. Misek D. E. Vinco M. Sanders D. (2005). Molecular Classification of Papillary Thyroid Carcinoma: Distinct BRAF, RAS, and RET/PTC Mutation-specific Gene Expression Profiles Discovered by DNA Microarray Analysis. Oncogene 24 (44), 6646–6656. 10.1038/sj.onc.1208822 16007166

[B9] Gong Y. Zou B. Chen J. Ding L. Li P. Chen J. (2019). Potential Five-MicroRNA Signature Model for the Prediction of Prognosis in Patients with Wilms Tumor. Med. Sci. Monit. 25, 5435–5444. 10.12659/msm.916230 31328722 PMC6668497

[B10] Harris A. M. Warner B. W. Wilson J. M. Becker A. Rowland R. G. Conner W. (2007). Effect of α1-Adrenoceptor Antagonist Exposure on Prostate Cancer Incidence: An Observational Cohort Study. J. Urol. 178 (5), 2176–2180. 10.1016/j.juro.2007.06.043 17870114 PMC2084470

[B11] Kennedy J. M. Robinson R. A. (2016). Thyroid Frozen Sections in Patients with Preoperative FNAs. Am. J. Clin. Pathol. 145 (5), 660–665. 10.1093/ajcp/aqw042 27124950

[B12] Ko Y. S. Hwang T. S. Kim J. Y. Choi Y. L. Lee S. E. Han H. S. (2017). Diagnostic Limitation of Fine-Needle Aspiration (FNA) on Indeterminate Thyroid Nodules Can Be Partially Overcome by Preoperative Molecular Analysis: Assessment of RET/PTC1 Rearrangement in BRAF and RAS Wild-type Routine Air-Dried FNA Specimens. Int. J. Mol. Sci. 18 (4). 10.3390/ijms18040806 PMC5412390 28417935

[B13] Li H.-Y. Grifone R. Saquet A. Carron C. Shi D.-L. (2013). The Xenopus Homologue of Down Syndrome Critical Region Protein 6 Drives Dorsoanterior Gene Expression and Embryonic axis Formation by Antagonising Polycomb Group Proteins. Development 140 (24), 4903–4913. 10.1242/dev.098319 24301465

[B14] Lin Y.-P. Wu J.-I. Tseng C.-W. Chen H.-J. Wang L.-H. (2019). Gjb4 Serves as a Novel Biomarker for Lung Cancer and Promotes Metastasis and Chemoresistance via Src Activation. Oncogene 38 (6), 822–837. 10.1038/s41388-018-0471-1 30177841

[B15] Liu G. Pang Y. Zhang Y. Fu H. Xiong W. Zhang Y. (2019). GJB4 Promotes Gastric Cancer Cell Proliferation and Migration via Wnt/CTNNB1 Pathway. Ott Vol. 12, 6745–6755. 10.2147/ott.s205601 PMC6708386 31692499

[B16] Misiakos E. P. Margari N. Meristoudis C. Machairas N. Schizas D. Petropoulos K. (2016). Cytopathologic Diagnosis of fine Needle Aspiration Biopsies of Thyroid Nodules. Wjcc 4 (2), 38–48. 10.12998/wjcc.v4.i2.38 26881190 PMC4733475

[B17] Muzza M. Colombo C. Pogliaghi G. Karapanou O. Fugazzola L. (2020). Molecular Markers for the Classification of Cytologically Indeterminate Thyroid Nodules. J. Endocrinol. Invest. 43 (6), 703–716. 10.1007/s40618-019-01164-w 31853887

[B18] Nylén C. Mechera R. Maréchal-Ross I. Tsang V. Chou A. Gill A. J. (2020). Molecular Markers Guiding Thyroid Cancer Management. Cancers (Basel) 12 (8). 10.3390/cancers12082164 PMC7466065 32759760

[B19] Rudzińska M. Grzanka M. Stachurska A. Mikula M. Paczkowska K. Stępień T. (2019). Molecular Signature of Prospero Homeobox 1 (PROX1) in Follicular Thyroid Carcinoma Cells. Int. J. Mol. Sci. 20 (9). 10.3390/ijms20092212 PMC6539481 31060342

[B20] Saito R. Smoot M. E. Ono K. Ruscheinski J. Wang P.-L. Lotia S. (2012). A Travel Guide to Cytoscape Plugins. Nat. Methods 9 (11), 1069–1076. 10.1038/nmeth.2212 23132118 PMC3649846

[B21] Schneider D. F. Chen H. (2013). New Developments in the Diagnosis and Treatment of Thyroid Cancer. CA Cancer J. Clin. 63 (6), 374–394. 10.3322/caac.21195 23797834 PMC3800231

[B22] The Lancet L. (2017). Thyroid Cancer Screening. The Lancet 389 (10083), 1954. 10.1016/s0140-6736(17)31349-1 28534740

[B23] Tschirdewahn S. Panic A. Püllen L. Harke N. N. Hadaschik B. Riesz P. (2019). Circulating and Tissue IMP3 Levels Are Correlated with Poor Survival in Renal Cell Carcinoma. Int. J. Cancer 145 (2), 531–539. 10.1002/ijc.32124 30650187

[B24] Tsuchiya Y. Mii Y. Okada K. Furuse M. Okubo T. Takada S. (2018). Ripply3 Is Required for the Maintenance of Epithelial Sheets in the Morphogenesis of Pharyngeal Pouches. Develop. Growth Differ. 60 (2), 87–96. 10.1111/dgd.12425 29471585

[B25] UniProt Consortium (2010). The Universal Protein Resource (UniProt) in 2010. Nucleic Acids Res. 38 (Suppl. l_1), D142–D148. 10.1093/nar/gkp846 19843607 PMC2808944

[B26] Wang K. Lü H. Qu H. Xie Q. Sun T. Gan O. (2019). miR-492 Promotes Cancer Progression by Targeting GJB4 and Is a Novel Biomarker for Bladder Cancer. Ott Vol. 12, 11453–11464. 10.2147/ott.s223448 PMC6935362 31920334

[B27] Xiong Y. Wang R. Peng L. You W. Wei J. Zhang S. (2017). An Integrated lncRNA, microRNA and mRNA Signature to Improve Prognosis Prediction of Colorectal Cancer. Oncotarget 8 (49), 85463–85478. 10.18632/oncotarget.20013 29156733 PMC5689623

[B28] Yu Y. Liu C. Zhang J. Zhang M. Wen W. Ruan X. (2017). Rtfc (4931414P19Rik) Regulates *In Vitro* Thyroid Differentiation and *In Vivo* Thyroid Function. Sci. Rep. 7, 43396. 10.1038/srep43396 28230092 PMC5322522

[B29] Zheng B. Liu J. Gu J. Lu Y. Zhang W. Li M. (2015). A Three-Gene Panel that Distinguishes Benign from Malignant Thyroid Nodules. Int. J. Cancer 136 (7), 1646–1654. 10.1002/ijc.29172 25175491

[B30] Zhong L. K. Gan X. X. Deng X. Y. Shen F. Feng J. H. Cai W. S. (2020). Potential five-mRNA S-ignature M-odel for the P-rediction of P-rognosis in P-atients with P-apillary T-hyroid C-arcinoma. Oncol. Lett. 20 (3), 2302–2310. 10.3892/ol.2020.11781 32782547 PMC7400165

